# EEG correlates of verbal and nonverbal working memory

**DOI:** 10.1186/1744-9081-1-20

**Published:** 2005-11-15

**Authors:** Grace Hwang, Joshua Jacobs, Aaron Geller, Jared Danker, Robert Sekuler, Michael J Kahana

**Affiliations:** 1 Volen Center for Complex Systems, Brandeis University, Waltham, MA, 02454, USA; 2 Department of Psychology, University of Pennsylvania, Philadelphia, PA, 19104, USA

## Abstract

**Background:**

Distinct cognitive processes support verbal and nonverbal working memory, with verbal memory depending specifically on the subvocal rehearsal of items.

**Methods:**

We recorded scalp EEG while subjects performed a Sternberg task. In each trial, subjects judged whether a probe item was one of the three items in a study list. Lists were composed of stimuli from one of five pools whose items either were verbally rehearsable (letters, words, pictures of common objects) or resistant to verbal rehearsal (sinusoidal grating patterns, single dot locations).

**Results:**

We found oscillatory correlates unique to verbal stimuli in the *θ *(4–8 Hz), *α *(9–12 Hz), *β *(14–28 Hz), and *γ *(30–50 Hz) frequency bands. Verbal stimuli generally elicited greater power than did nonverbal stimuli. Enhanced verbal power was found bilaterally in the *θ *band, over frontal and occipital areas in the *α *and *β *bands, and centrally in the *γ *band. When we looked specifically for cases where oscillatory power in the time interval *between *item presentations was greater than oscillatory power *during *item presentation, we found enhanced *β *activity in the frontal and occipital regions.

**Conclusion:**

These results implicate stimulus-induced oscillatory activity in verbal working memory and *β *activity in the process of subvocal rehearsal.

## Background

Evidence from studies of working memory has long indicated that the rate at which items are encoded into memory and subsequently recognized varies with stimulus type [1-3], that different processes are involved in verbal and nonverbal working memory [4,5], and that verbal memory depends specifically on the subvocal rehearsal of items [6,7]. Evidence from magnetoencephalography (MEG), scalp EEG, and intracranial EEG studies has implicated oscillations in both verbal and nonverbal working memory [8-27]. In some studies, oscillations were sustained from the time an item was presented until it was later tested [12,17]. In other studies, oscillations were linked to the retention interval, with oscillatory amplitude or coherence increasing with memory load [10,11]. Across these studies, oscillations associated with working memory were reported in the 4–8 Hz *θ *band [10,14,15,17,19,20,25], the 9–12 Hz *α *band [9,11,16,18], the 14–28 Hz *β *band [20,21,23] and the 30–60 Hz *γ *band [12,19,23,24,26,27]. Building on the abundance of research linking oscillations and working memory, we attempted to identify oscillatory correlates of verbal memory, and particularly the correlates of subvocal rehearsal. We addressed this aim by examining the oscillatory correlates of working memory for a range of stimuli that were designed to vary widely in the extent to which they could be verbally rehearsed.

**Figure 1:**
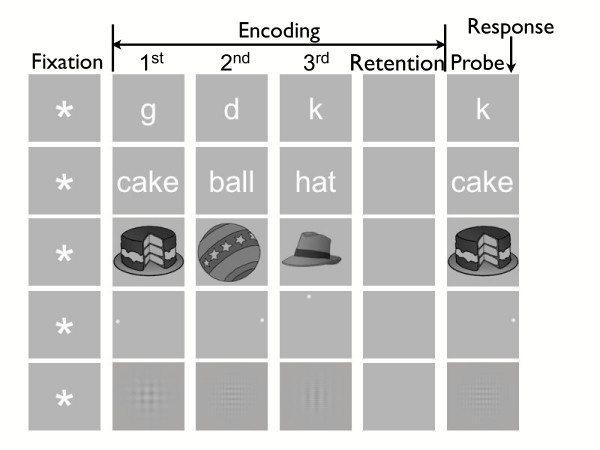
**Sternberg Paradigm**. Schematic illustrating one trial of each stimulus pool in the Sternberg task: letter, word, object, spatial, grating. Also shown are stimulus class definitions. The time interval from the moment the subject depressed the advance key until the onset of the fixation stimulus was 400 ms; we therefore used this 400-ms interval as our **baseline **stimulus class (not labeled). The **fixation **stimulus class (labeled *Fixation*) covers the time interval starting at the presentation of an asterisk and ending just before the presentation of the first study item. The **encoding **stimulus class (labeled *Encoding*) is the time interval covering the presentation of the 3 study items (labeled *1*^*st*^, *2*^*nd*^, *3*^*rd*^) including the 500-ms interval following the disappearance of the third study item (labeled *Retention*). The time interval covering the start of the presentation of the first item and ending with the presentation of the third item is referred to as the study interval. The encoding stimulus class therefore contains two intervals: the study interval followed by the retention interval. The **probe **stimulus class (labeled *Probe*) is the time interval starting with the onset of the probe stimulus and ending with the subject's response. The **response **stimulus class started with the onset of subject response and ended 1 sec later, well before the start of the next trial (not labeled).

EEGs were recorded from subjects while they performed a variant of the Sternberg task [28], a prototypical and widely used test of working memory. Subjects were shown a set of 3 study items to hold in memory over a short retention interval. This retention interval was followed by a probe item. Subjects were asked to indicate, as quickly and accurately as possible, whether the probe item was a *target *(an item present in the study list) or a *lure *(an item absent from the study list). Study lists were composed of stimuli drawn from each of five pools. As illustrated in Figure 1, these stimuli were letters, words, namable objects, single dot (i.e., spatial) positions, and sinusoidal grating patterns. Whereas subjects could easily rehearse lists of letters, words, and namable objects, spatial positions and sinusoidal grating patterns were difficult for subjects to name and rehearse. Hereafter, we refer to the letter, word, and object stimuli collectively as the *verbal *stimulus type, and we refer to the spatial and grating stimuli collectively as the *nonverbal *stimulus type. We hypothesized that studying differential neuroelectrical responses across these stimulus types and across successive phases of the viewing cycle would isolate distinct neural processes that correlate with subvocal rehearsal.

Our central question was whether differences in oscillatory power reflect the verbal rehearsal process. Although this question was difficult to answer, because there was no way to determine precisely when subjects were rehearsing, we could make certain inferences about subvocal rehearsal from our understanding of the stimuli themselves. First, we had reason to expect that subvocal rehearsal would be greater for verbal, namable items than for nonverbal items, such as dot locations or sinusoidal gratings. This enabled us to predict that there would be a systematic difference in overall oscillatory power between verbal and nonverbal stimulus types (Criterion 1). Second, we expected that rehearsal of verbal stimuli would be more likely to occur during interstimulus intervals (ISI) – that is, between item presentations or between the last item and the test probe – than during stimulus-presentation intervals (SPI) when stimuli are in view. Consistent with previous observations that showed *increases *in power to be correlated temporally with encoding [12,15,17] and retention [17,23], we predicted that power due to verbal rehearsal during ISI would be greater than power during SPI (Criterion 2). Combining these two criteria, we investigated oscillatory power across stimulus types and across successive phases of the viewing cycle (i.e., ISI vs. SPI).

## Results

### Behavioral Data

**Figure 2:**
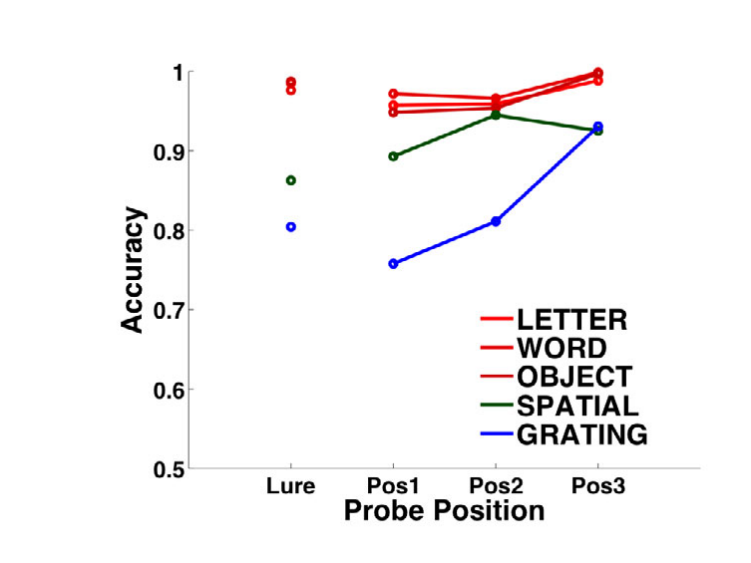
**Performance Accuracy**. Performance accuracy is plotted for each of the five stimulus pools as a function of probe position across all subjects (*n *= 12). Probes that did not appear in the study list are labeled *Lure*, while probes that were part of the study list are labeled by their position (i.e., *Pos*1 refers to probes that appeared first in a study list).

As shown in Figure 2, mean accuracy was highest for the verbal stimuli (i.e., letter, word, and namable objects) and lowest for the sinusoidal gratings. As expected based on previous studies [29,30], accuracy for the verbal and grating stimuli was highest for targets that replicated the most recently presented list item. Consistent with these trends, a 5 (stimulus pool) × 4 (probe position) ANOVA on accuracy revealed statistically significant main effects of stimulus pool (*F*(4, 44) = 99, MSe = 0.0021, *p *< 0.001) and probe position (*F*(3,33) = 9.4, MSe = 0.0048, *p *< 0.001), as well as a significant interaction between these factors (*F*(12,132) = 4.9, MSe = 0.0027, *p *< 0.001). Reaction times (RTs) across stimuli also varied, with verbal stimuli eliciting the shortest RTs and nonverbal stimuli eliciting the longest RTs. Since we did not vary the length of study lists, accuracy and RTs were not analyzed as a function of list length.

### Electrophysiological Data

**Figure 3:**
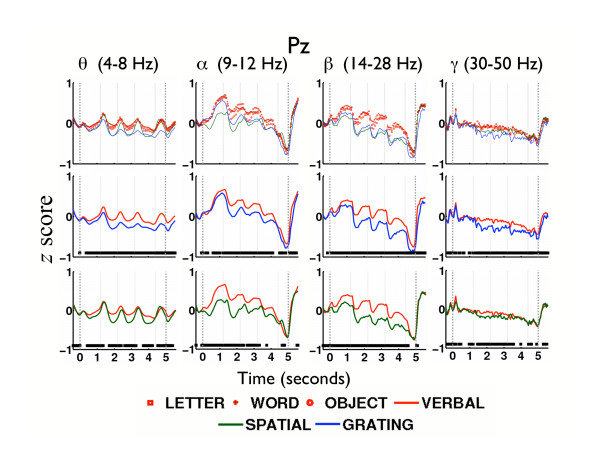
**Power Time Curves**. Z-transformed wavelet power in the *θ*, *α*, *β*, and *γ *frequency bands are illustrated in the parietal brain region (from electrode Pz) for 12 subjects. The fixation asterisk appeared at t = 0 sec, as indicated by the vertical dashed line. The 3 study items appeared at average times of 1.2 sec, 2.2 sec, and 3.2 sec as indicated by vertical dotted lines. The retention interval started at average time t = 3.9 sec. The probe item appeared at average time t = 4.4 sec (vertical dotted line), and the response was made on average at t = 5 sec (vertical dashed line). The first row illustrates power for each of the five stimulus pools. The second and third rows illustrate power in the verbal-vs-grating and in the verbal-vs-spatial tasks, respectively. Verbal refers to the mean power from the letter, word, and object tasks. Significant differences between verbal and each of the nonverbal tasks (*t*(11) = 2.2, *p *< 0.05) are denoted by a thick horizontal bar at the bottom of each panel in the second and third rows.

Figure 3 shows the time course of mean Z-transformed oscillatory power at electrode Pz across fixation, encoding, retention, and probe intervals. From left to right, the upper panels show time courses at each of the four frequency bands. Within each panel of Figure 3 (top row), time courses of oscillatory power are seen to be quite similar across the three verbal stimuli (letter, word, object denoted by red symbols) but dissimilar from both the spatial (green) and grating (blue) stimuli. Because we did not observe any systematic differences among the three verbal stimuli, and because analyses of other electrodes exhibited qualitatively similar time courses to those shown in Figure 3 (upper panel), we combined data across verbal stimuli for all subsequent analyses. We treated the two nonverbal stimuli separately because they exhibited systematic differences in several of our analyses, as described in greater detail below. The middle and lower panels of Figure 3 compare the time courses of oscillatory power for verbal-vs.-spatial stimuli and verbal-vs.-grating stimuli, respectively. Significant differences for each of these comparisons are denoted by a thick horizontal bar on the bottom of each panel (*t*(11) = 2.2, *p *< 0.05).

During the encoding interval, the amplitude of power in *θ*, *α*, and *β *exhibited responses that were induced by the onset of the stimulus (see Figure 3, columns 1–3). *θ *showed stimulus-induced enhancement: that is, *θ *power increased and decreased sharply in each SPI (Figure 3, column 1). *α *and *β *showed stimulus-induced reduction: that is, *α *and *β *power decreased during the SPI and increased again during the ISI (Figure 3, columns 2 and 3). During the probe interval, *α *and *β *power declined steadily to a minimum and immediately returned to above-baseline levels following the responses. *γ *power showed a slight systematic decline that was insensitive to the timing of study-item presentations, with a rapid return to baseline levels immediately following the responses (Figure 3, column 4). Power in all bands was generally lower for grating stimuli than for spatial stimuli, both of which were exceeded by verbal stimuli, with the largest difference in power seen in *θ *and *β *(Figure 3, columns 1 and 3). This consistent pattern of higher oscillatory power elicited by verbal stimuli indicates that oscillatory activity increases during the processing of verbally rehearsable stimuli. In addition, the overall decline in *α*, *β*, and *γ *power over the course of the trial for all stimulus types, suggests a generalized desynchronization of oscillations with increased memory load.

### Criterion 1. Verbal versus Nonverbal Stimulus Types

**Figure 4:**
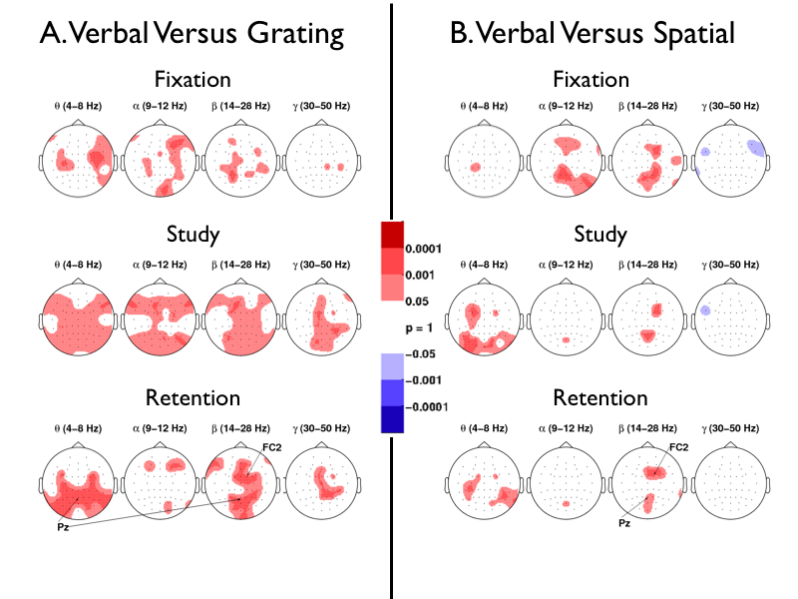
**Topographic Analysis of Stimulus Types**. Brain regions that show enhanced verbal power and enhanced nonverbal power are illustrated in (a) Verbal-vs.-Grating, and (b) Verbal-vs.-Spatial during the fixation, study, and retention intervals. Red means the verbal stimuli elicited significantly more mean power than did nonverbal stimuli during each of the respective intervals. Blue means nonverbal stimuli elicited significantly more mean power than did verbal stimuli during each of the respective intervals. Significance was determined by a randomization test on matched pairs, with a Type I error rate of 0.001, which corresponds to 0.24 electrodes that could have shown significance by chance across all subjects (randomization test, *p *< 0.001, *df *= 11). Multiple comparisons for the number of electrodes and frequency bands were accounted for with a resampling method on matched pairs (see section on Randomization Test Procedure). Topographic visualization was done with the topoplot function in the EEGLAB Matlab toolbox [56].

To determine whether power elicited by verbal stimuli differed significantly from power elicited by spatial and grating stimuli, we conducted a topographic analysis, as shown in Figures 4a and 4b. We refer to instances where verbal tasks elicited significantly greater power than did nonverbal tasks as *enhanced verbal power *(EVP; randomization test, *p *< 0.001, *df *= 11). Similarly, instances where nonverbal tasks elicited significantly greater power than did verbal tasks are referred to as *diminished verbal power *(DVP; randomization test, *p *< 0.001, *df *= 11). Figures 4a and 4b illustrate the topographic distribution of EVP and DVP for the comparison of *verbal-vs.-grating *(VG) stimuli and the comparison of *verbal-vs.-spatial *(VS) stimuli, respectively. Within each panel, EVP and DVP are illustrated separately for the fixation (top row), study (middle row), and retention intervals (bottom row).

In the fixation interval, EVP was sparsely distributed in all frequency bands in the VG comparison (Figure 4a, top row); EVP was observed centrally in *θ*, and in frontal and parietal areas in *α *and *β *in the VS comparison (Figure 4b, top row). Sparsely distributed DVP was observed in *γ *in only the VS comparison (Figure 4b, top row, column 4).

In the study interval, EVP occurred bilaterally in *θ*, *α*, and *β*, and centrally in *γ *in the VG comparison (Figure 4a, middle row). For the VS comparison, we found EVP in *θ *distributed in frontal and occipital areas, EVP in *α *localized to one parieto-occipital site, EVP in *β *distributed in frontal and parietal areas (Figure 4b, middle row), and DVP in *γ *localized to one left frontal site (Figure 4b, middle row, column 4).

During the retention interval, the VG comparison revealed that EVP in *θ *was distributed bilaterally in the parieto-occipital regions (Figure 4a, bottom row, column 1), EVP in *α *occurred sparsely in bilateral regions (Figure 4a, bottom row, column 2), EVP in *β *occurred bilaterally along the midline (Figure 4a, bottom row, column 3), and EVP in *γ *occurred centrally (Figure 4a, bottom row, column 4). The VS comparison showed that EVP in *θ *was distributed bilaterally in the centro-parietal regions (Figure 4b, bottom row, column 1), EVP in *α *was localized to one parieto-occipital site (POz; Figure 4b, bottom row, column 2), and EVP in *β *occurred in the frontal and parieto-occipital areas (Figure 4b, bottom row, column 3). We did not observe EVP in the *γ *band.

**Figure 5:**
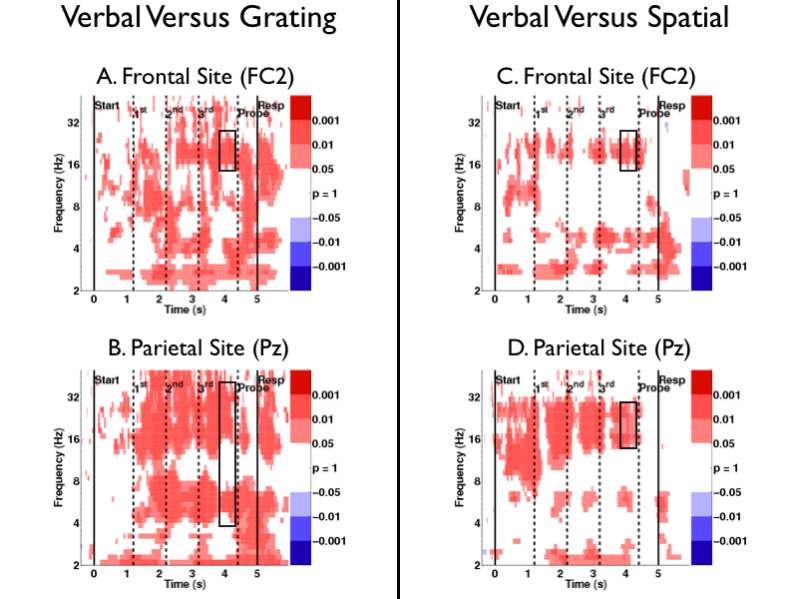
**Time-Frequency Spectrogram**. Time-frequency representations of *p *values in the verbal-vs.-grating comparison are shown in (a) from a frontal site (electrode FC2), and in (b) from a parietal site (electrode Pz). Time-frequency representations of *p *values in the verbal-vs.-spatial comparison are shown in (c) from a frontal site (electrode FC2), and in (d) from a parietal site (electrode Pz). Frequency bands of interest in the retention interval are boxed in black. Red means the verbal stimuli elicited significantly more power than did nonverbal stimuli. Blue means nonverbal stimuli elicited significantly more power than did verbal stimuli. Significance was determined by a randomization test on matched pairs, with a Type I error rate of 0.01, which corresponds to 53 time bins that could have shown significance by chance across all subjects (randomization test, *p *< 0.01, *df *= 11). Multiple comparisons for the number of electrodes and frequency bands were accounted for with a resampling method on matched pairs (see section on Randomization Test Procedure).

To understand the relation between topographic significance during the task interval and consecutive time bins at which EVP was present, we further explored the temporal characteristics of EVP. Hence we present time-frequency plots from frontal and parietal regions for both the VG and VS comparisons, first from a right centro-frontal site (FC2) and then from a midline parietal site (Pz). For the VG comparison, Figure 5a shows a temporally persistent EVP in *β *at electrode FC2 during the retention interval (which started at approximately 3.9 sec and ended at approximately 4.4 sec). Similarly, Figure 5b shows a continuously persistent EVP in *θ*, *β*, and 32-Hz *γ *at electrode Pz during the retention interval. Both Figures 5a and 5b fail to show EVP in *α *during the retention interval, which is consistent with the topographic analysis shown in Figure 4a (bottom row, column 2). For the VS comparison, Figure 5c shows temporally persistent EVP in *β *at electrode FC2 during the retention interval. Similarly, Figure 5d shows EVP in *β *at electrode Pz during the retention interval. Both Figures 5c and 5d fail to show EVP in *α *and *γ *during the retention interval, which is consistent with the topographic results shown in Figure 4b (bottom row, columns 2 and 4).

### Criterion 2. Comparison of Viewing-Cycle Phases (ISI vs. SPI)

**Figure 6:**
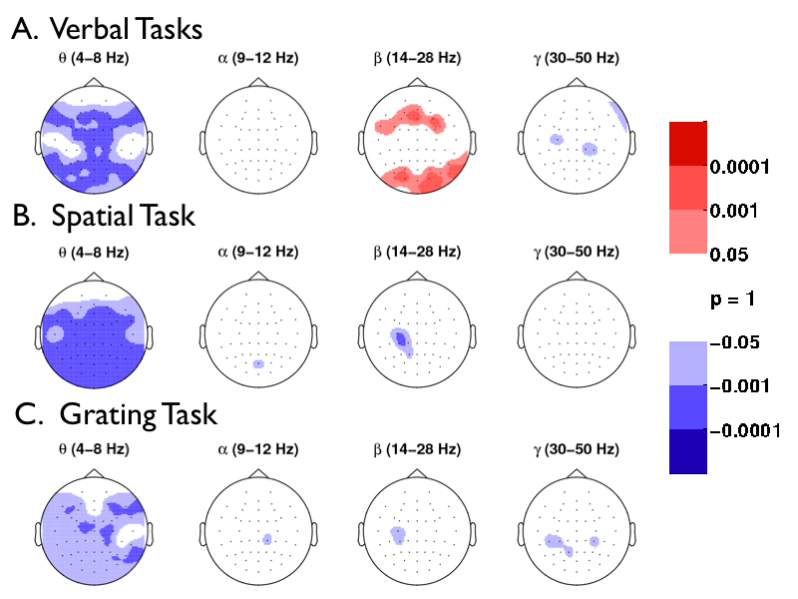
**Topographic Analysis of Phases of the Viewing Cycle: stimulus presentation interval (SPI) versus (ISI) interstimulus presentation interval**. Brain regions that differ significantly in power levels between SPI and ISI are illustrated. (a) Verbal (mean of letter, word, and object), (b) Spatial, and (c) Grating. Red means that power during ISI was significantly greater than power during SPI; blue means that power during SPI was significantly greater than power during ISI. Significance was determined by a randomization test on matched pairs, with a Type I error rate of 0.001, which corresponds to 0.24 electrodes that could have shown significance by chance across all subjects (randomization test, *p *< 0.001, *df *= 11). Multiple comparisons for the number of electrodes and frequency bands were accounted for with a resampling method on matched pairs (see section on Randomization Test Procedure).

We used a randomization procedure to determine whether ISI power was greater than SPI power in the verbal, spatial, and grating tasks (see Figures 6a–c). As shown in Figure 6a (column 3), only the *β *band from verbal tasks elicited significantly more ISI power than SPI power over the frontal and occipital areas (randomization test, *p *< 0.001, *df *= 11). In contrast, nonverbal tasks did not elicit more ISI power than SPI power at any electrode or frequency band (Figures 6b and 6c). Figures 6a–c (column 1) also show that power during SPI was greater than power during ISI in *θ *for both verbal and nonverbal stimulus types (randomization test, *p *< 0.001, *df *= 11).

## Discussion

### Summary of Criterion 1. Verbal versus Nonverbal Stimulus Types

Overall, we observed EVP across the fixation, study and retention intervals (see Figure 4). Because we employed a blocked paradigm, subjects were aware of which stimulus pool to expect in successive trials. Therefore, EVP during the fixation interval may reflect preparatory processes that are unique to (or relatively more prominent in) the verbal tasks. EVP during the study and retention intervals may reflect any aspect of information processing that differentiates verbal and non-verbal stimuli. One candidate process is subvocal rehearsal, which subjects actively use for verbal materials but which is almost impossible with the nonverbal stimuli used in our study. It is also possible, however, that EVP reflects the greater ease of encoding and retaining verbal items. We will return to this issue later in the discussion.

The differing EVP topographies between the VG and VS comparisons during the study and retention intervals indicate that grating stimuli attenuated *α *oscillations more than spatial stimuli did (see Figures 4a and 4b, second and third rows, column 2). We offer two possibilities that could give rise to these different *α *EVP topographies. First, we suggest that *α *oscillations could play differential roles in the spatial and grating tasks. *α *oscillations could be enhanced by the strategies that subjects used to spatially encode items that appeared close to their peripheral field-of-view while they simultaneously attempted to suppress eye movements. An alternative explanation can be cast in terms of attentional demands: in ratings along a continuum of task difficulty obtained from our strategy questionnaire, the verbal tasks rated much easier than the spatial task, and the spatial task was in turn easier than the grating task. If we take the common view that *α *oscillations index task-related attentional demands [13], and if we posit that subjects allocated the most attention to the hardest tasks, then the EVP observed in the VG comparison could directly reflect differences in attentional demand between the verbal and grating tasks. Similarly, the lack of EVP observed in the VS comparison could suggest that the difference in attentional demand, as reflected by the amplitude of *α*, did not reach significance.

### Summary of Criterion 2. Comparison of Viewing-Cycle Phases

All stimulus types elicited significantly more SPI *θ *power than ISI *θ *power (see Figures 6a–c, column 1); this finding was consistent with the patterns of stimulus-induced *θ *increase observed in Figure 3 (column 1). These observations suggest that *θ *may be involved with perceptual and cognitive processes common to both verbal and nonverbal tasks while showing more power in response to the verbal tasks. In contrast, verbal tasks elicited significantly more ISI power than SPI power exclusively in the *β *frequency band (Figure 6a, column 3), thus showing that *β *frequency activity dissociates working memory for verbal and noverbal stimuli. Subvocal rehearsal is one candidate process that is responsible for this dissociation. Alternatively, differences in *β *may be a consequence of the greater difficulty associated with encoding and maintaining the nonverbal items.

### Roles of *β *Oscillations in Working Memory

Recalling that EVP in *β *was consistently observed during the retention interval in the VS and VG comparisons along the midline frontal and parietal areas (Figures 4a and 4b), and that ISI power was greater than SPI power in *β *(Figure 6, first row, column 3), we reason that these data provide functional evidence that *β *oscillations support an important process in subvocal rehearsal. Although *β *oscillations have often been associated with muscle movement artifacts [31], *β *effects observed in our dataset were not localized along the temporal regions (on the scalp) known to be involved in finger movements. Furthermore, prior research associated sustained *β *oscillations with the retention of stimuli [23,24,32]. In a delayed-match-to-sample study that used stimuli composed of abstract shapes, *β *oscillations increased and remained elevated during the retention interval [23] at a frontal (Fz) and an occipital site (POz) along the midline. However, these authors did not assess the effect of stimulus-rehearsability on *β *activity. We therefore can only speculate, extrapolating from observations in our study, that *β *oscillations induced by verbal stimuli, if verbal stimuli had been tested, might also have been sustained during the retention interval, as were those elicited by abstract shapes. In our study, verbal stimuli elicited larger and more sustained *β *oscillations than did nonverbal stimuli during the retention interval (as depicted in Figure 3, column 3).

### Roles of Oscillations in Verbal and Nonverbal Memory

To identify oscillatory and topographical correlates of rehearsal, a number of studies have specifically compared verbal and nonverbal stimulus types. In a stimulus-reproduction task, oscillations in the *θ *and *γ *bands were shown to exhibit levels of increased synchrony between the posterior association cortex and the prefrontal cortex during the retention interval [19]. The degree of synchrony did not distinguish abstract stimuli from verbal stimuli, however. In a Sternberg task, increased power in *θ *and *γ *during the retention interval was shown to be synchronous in the left frontal area between electrode pairs Fz and Fpl for verbal stimuli but not for irregular rectangular stimuli [20]. This finding was consistent with the idea that the left prefrontal cortex is involved in verbal processing. An n-back task, which required subjects to identify both letters (verbal condition) and their spatial position (visual condition), revealed that the visual condition attenuated the amplitude of upper *α *oscillations in the right hemisphere, while the verbal condition did not [9]. This finding suggests that working memory for verbal information is less dependent on the right posterior cortex than working memory required for visual information, a distinction that is consistent with the idea that visual processing occurs in the right hemisphere. Most of these studies [9,20] have successfully used attributes of oscillations (i.e., levels of synchrony, fluctuation in amplitude) to link verbal processes with the left hemisphere and visual processes with the right hemisphere. We did not find a hemispheric dissociation between verbal and nonverbal memory; instead, we found oscillatory effects of stimulus types distributed bilaterally in both *θ *and *β *frequency bands.

### Neuroimaging Evidence in Verbal Working Memory

Evidence from PET and fMRI studies, coupled with support from lesion studies [33-35], strongly suggests that distinct neural substrates subserve each of the two subsidiary systems of working memory: the phonological loop (verbal memory) and the visuospatial sketchpad (visual memory) [4,5]. In these studies, mental processes that require subvocal rehearsal preferentially activated the left prefrontal cortex, the bilateral occipital cortex [36], Broca's region, the premotor cortex, the supplemental motor areas, the left posterior parietal cortex [37], and the cerebellum [38,39]. In contrast, processes requiring the use of visual memory preferentially activated the right dorsal prefrontal cortex, the right parietal cortex, and the right middle frontal gyrus [36,40].

We observe caution in making claims about a possible correlation between our results based on scalp EEG signals and any results obtained by neuroimaging. In this study, we found that verbal stimuli generally elicited greater oscillatory power than did nonverbal stimuli during the study, retention and retrieval phases of the Sternberg task. When we further compared oscillatory power in the stimulus-presentation and interstimulus intervals, we found that *β *power was significantly higher during the stimulus-presentation interval, but only for the verbal tasks. This pattern, which was not found at other frequencies, indicates that *β *covaries either with rehearsal or some other aspect of information processing that differs between verbal and nonverbal stimuli. Wherease this *β *effect was seen bilaterally, several neuroimaging studies have linked the left prefrontal area to the process of subvocal rehearsal [5,41]. It should be noted, however, that numerous studies employing verbal tasks have shown activations bilaterally in both hemispheres [39,41-43] during the task-maintenance interval (akin to our retention interval). One study attributed frontal right-hemispheric activations to the process of updating the central executive [44,45] with verbal information [46]. Because the scalp EEG technique lacks fine spatial resolution, it was not possible in our experiment to disentangle subvocal rehearsal from other aspects of executive function. At the present time, the relation between scalp-recorded oscillations and hemodynamic responses measured using fMRI remains largely unknown, however recent evidence from intracranial recordings suggests a link between locally-generated gamma oscillations and increased hemodynamic activity [47]. Future studies that combine scalp EEG and fMRI recordings during working memory tasks may help to resolve these open issues.

## Conclusion

We investigated oscillatory power across stimulus types and across phases of the viewing cycle, considering only those brain regions satisfying Criterion 1 (verbal power differs from nonverbal power) and Criterion 2 (ISI power is greater than SPI power) to be correlated with subvocal rehearsal. Our results indicate that stimulus-induced oscillatory activity is involved in verbal working memory, with verbal stimuli eliciting more power than nonverbal stimuli in *θ*. The mean amplitude of *θ *power during ISI was not greater than the mean *θ *power during SPI, thus suggesting that *θ *oscillations are involved with perceptual and memory encoding processing common to both verbal and nonverbal tasks. In contrast, *β *oscillations simultaneously satisfied Criteria 1 and 2 along the midline at frontal and parietal brain regions, thereby distinguishing verbal stimuli from nonverbal stimuli across stimulus types and across phases of the viewing cycle. These results thus implicate *β *oscillations in the subvocal rehearsal process of verbalizable items.

## Methods

### Subjects

Subjects were 12 right-handed volunteers ranging in age from 19 to 29. Eight were male and four were female. All subjects had normal or corrected-to-normal vision. All subjects gave informed consent to a protocol reviewed and approved by the Brandeis University Committee for the Protection of Human Subjects. Subjects were given a base payment plus bonus payments proportional to their performance. Subjects participated in a total of five sessions, each of which was conducted on a different day.

### Procedure

Five pools of different stimuli were employed: single *letters*, pictures of *objects *from the Snodgrass [48] picture set, one-syllable *words *corresponding to the object pool, dots that appeared at different (*spatial*) positions on the monitor, and sinusoidal patterns (*grating*). Each stimulus-type pool contained 16 stimuli. We considered a stimulus verbal if it was amenable to subvocal rehearsal, and nonverbal if it resisted subvocal rehearsal. Hence, letters, words, and objects were all considered verbal because of the ready availability of tags for rehearsal (e.g., the word *car *for the corresponding object), whereas spatial and grating stimuli were considered nonverbal. A sample of each stimulus pool is shown in Figure 1.

Each subject was tested with stimuli from each of the five different stimulus pools (letter, word, object, spatial, grating). Stimuli were shown on a computer monitor positioned 57 cm away from the subject. The approximate visual angle for the letter, word, object, and grating stimuli was 5°; for the spatial stimuli, it was 10°. Every trial of the experiment was self-paced; subjects initiated a trial by pressing an advance key; this was followed by a 400-ms interval before the onset of a fixation cue (an asterisk). Each trial started with a fixation cue centered in the middle of the computer monitor for a duration of 1 sec (± 200 ms jitter), followed by a study set of 3 stimuli. Each stimulus was shown on the monitor for 700 ms, followed by a 275 ms ± 75 ms ISI; the ISI was randomly jittered serving to decorrelate physiological responses which may occur due to successive stimuli presentations. After the offset of the third stimulus, a short retention interval (500 ms ± 75 ms jitter) was followed by a fourth item (*probe*) for 750 ms. Subjects were preinstructed to determine as quickly and accurately as possible after probe onset whether the probe item was part of the preceding study list. In order to obtain the fastest reaction times possible, they were also preinstructed to respond to a *target *by pressing the right control key (dominant hand); they were to respond to a *lure *by pressing the left control key (nondominant hand). A blink break followed each response, and subjects were prompted to continue at their own pace by pressing the down-arrow key. To prevent potential interference between a completed trial and the start of the next trial, a minimum of 1.5 sec was preprogrammed to pass before the next trial could begin. Feedback on accuracy and response time was given at the end of each block of trials.

### Stimulus Description

The letter pool contained the following letters: b, c, d, f, g, h, j, k, 1, m, n, p, q, r, t, and v. The object pool contained pictures of the following nouns: ball, bat, bed, bell, cake, car, chair, dog, ear, fly, fork, hat, heart, key, kite, and shoe. Each word in the word pool corresponded exactly to each object in the object pool. The spatial pool contained presentations of a solid white circle 1 cm in diameter (dot) at 16 nonoverlapping locations along the circumference of an invisible circle (10 cm in diameter) that was centered on the computer monitor. This configuration ensured that all dots fell close to the subjects' peripheral vision. Because there were 16 fixed dot positions, these positions were not easily encoded as clock-face positions (e.g., three o'clock). The grating pool contained two-dimensional textures, similar to those used in prior studies [49,50]. Each stimulus was a superposition of one horizontal and one vertical sinusoidal luminance grating, generating a luminance profile described by



where *L*_*avg *_represents mean luminance (L); *f *and *g *represent the spatial frequency of the vertical and horizontal components, respectively; and *A *is defined by



Parameters used to generate the 16 grating stimuli were *A *= 0.25, . The luminance of the monitor was linearized by means of calibration routines from Brainard & Pelli's Psychtoolbox [51].

### Stimulus-presentation Constraints

There were 10 blocks per session, 2 blocks for each of the 5 stimulus pool, for a total of 300 trials per session. Across all 5 sessions there were thus 1500 trials, 300 trials per stimulus pool. Each block comprised 15 targets and 15 lures. The target trials, in turn, probed each study position with equal probability. Study items in the current trial could not be from study items in the preceding two trials. Similarly, probe items in the current trial could not be from the preceding two trials. Further, a study item was allowed to be a lure only once in each block. Finally, the sequence of target trials and lure trials was randomized.

### EEG Recording

During EEG recording, subjects were instructed to remain silent and to minimize all body and eye movements (particularly blinking). Because the study was self-paced, subjects were also encouraged to take as many breaks between trials as they needed to maintain their concentration and optimize their performance. During the entire study, an experimenter quietly monitored the session from the back of the testing room. Lighting in the room was maintained at a constant level at all times. Recordings were obtained from 60 tin electrodes located in standard electrode positions embedded in an elastic cap (ElectroCap). EEG signals were amplified 10,000 times (Sensorium EPA6, 1 GΩ input impedance) with band limits between 0.03 and 50 Hz (12 dB/octave). Analog-to-digital signal conversion was implemented with a 12-bit data acquisition card (National Instrument PCI-6071E) with ± 5 *V *dynamic range. The overall system resolution was therefore 0.24 *μ*V/bit. Raw data was digitized at 256 Hz, well above the Nyquist minimum data sampling limit for our frequency region of interest (i.e., 2–50 Hz). Amplified signals were then digitally notch-filtered between 59 and 61 Hz to minimize 60-Hz line noise.

Electrode impedances were brought to < 50 kΩ, and interelectrode impedances were within 20 kΩ; skin impedances (ground and reference) were kept below 10 kΩ. Any electrodes that exhibited poor electrical characteristics were disconnected. All EEG signals were recorded referentially using the right mastoid (or right ear lobe). EEG signals were digitally re-referenced to the average EEG signal recorded from all electrically sound electrodes. Only signals recorded from low-impedance (< 50 kΩ) and electrically sound electrodes were included in the re-referencing. On any given session, no more than 5% of all electrodes had poor electrical contact and/or high impedances.

Six tin disc-electrodes were used to monitor electro-oculogram (EOG) activity. Vertical eye movements were isolated with electrodes positioned above and below each eye. Horizontal eye movements were isolated with electrodes placed at the lateral canthus of each eye. Each pair of EOGs was recorded bipolarly. Raw signals from EOG were used to detect blinking and automatic eye movements following Net Station's^™ ^weighted running-average algorithm [52]. If any one pair of EOG exceeded the combined rejection threshold of |100 *μ*volts|, the event (e.g., first study item presentation) that corresponded to the EOG spike was excluded from analysis. Fewer than 6% of all trials were excluded.

### Subject Questionnaire

At the conclusion of the final study session, subjects were asked to complete a strategy questionnaire. In section 1, subjects reported the strategies they used across study sessions as well as the relative effectiveness of those strategies. In section 2, subjects rated, on a 5-point scale, how often they used visual imagery and verbal labels to complete each of the five tasks (1 = never, 5 = always). Finally, in section 3, subjects rated the difficulty of each task relative to the other tasks on a continuum from 1 to 5 (1 = easiest, 5 = hardest).

### Behavioral Data Analysis

Accuracy and reaction times (RTs) were recorded for each trial. Trials with RTs shorter than 200 ms and greater than 1300 ms were excluded from analysis; this amounted to 2.1% of all trials.

### Oscillatory Power Analysis

Analyses were done separately for each stimulus pool and all stimulus classes (see Figure 1 for stimulus class definition). For each correctly answered trial (200 ms ≤ RT ≤ 1300 ms), oscillatory power was calculated by transforming the raw EEG signals with a 6-cycle Morlet wavelet [53] in logarithmically spaced frequencies between 2 and 50 Hz (38 intervals: 2^*x*/8 ^Hz, for *x *∊ {8...45}). Due to random temporal jitter in the ISI, stimulus class durations differed slightly across trials. To overcome this temporal variability, we applied a classical binning technique that accounted for intersubject and intertrial variance [54] by resampling stimulus classes into 20 equal length time bins; because the encoding stimulus class spanned the presentation of 3 study items plus the retention interval, the encoding class was resampled into 60 time bins. To minimize the variations in power across subjects and across trials, Z-transformed log_10 _wavelet power was computed for each frequency band, stimulus pool, subject, and stimulus class, following



where *Z *denotes Z-transformed power (Zpower, unitless); *P *denotes log_10 _wavelet power (dB); *t *denotes time bin (bin); *B *denotes time bins in the baseline stimulus class, which corresponds to 400 ms before and to the onset of the fixation stimulus class, |*B*| denotes the number of items in vector B; *σ*_*NB *_denotes standard deviation across trials calculated using the mean power from the baseline stimulus class (dB), and *N *denotes the total number of trials selected in a given condition. For a given frequency band, the Z-transform indicates how many standard deviations the average signal changed with respect to the baseline interval.

### Randomization Test Procedure

Signals in our EEG dataset were not independent; therefore, in order to account for multiple comparisons (across electrodes and frequency bands) and deviations from normality, we used a nonparametric randomization approach. Because we had equal electrode coverage across subjects and employed a repeated measures design, we were able to perform a randomization procedure on paired samples [55] at every electrode. That is, when we searched for stimulus-type-specific effects (e.g., verbal vs. grating), the first condition referred to the mean power in all verbal trials, the second condition referred to the mean power in all grating trials, and both conditions were calculated from the same time interval (e.g., encoding). In contrast, when we searched for time-interval-specific effects, the first condition referred to the mean ISI power, and the second condition referred to the mean SPI power, and both conditions were calculated from the same stimulus type (e.g., verbal). To account for multiple comparisons, we first generated one empirical distribution, using difference values across conditions at every electrode and frequency band, and then we combined data from all frequency bands and all electrodes into one large dataset. We used this this dataset to create 10,000 pseudodistributions by assigning opposite signs (+1, -1) to each difference value with equal probability over the 10,000 iterations. This was followed by a paired *t *test for every iteration of the pseudodistribution and then sorting the 10,000 *t *scores in order of increasing magnitude. Finally, we set the Type I error rate to 0.001, which equated to 0.24 electrodes that could have been significant by chance (0.001 ×  × 4 frequency bands = 0.24 electrodes). The *t *score from the pseudopopulation that corresponded to this Type I error rate was compared to that obtained from the original dataset at a given electrode and frequency band; if the *t *score from the original dataset was more significant than the *t *score generated by the pseudopopulation, we considered the original dataset to be significant (randomization test, *p *< 0.001, *df *= 11).

Similarly, when we generated time-frequency plots, in order to correct for multiple comparisons (across electrodes, frequency bands, and time bins) and deviations from normality, we used the same randomization procedure described above with two modifications. We added one additional dimension to represent time bin, and we randomized power at the level of discrete frequencies instead of taking the mean power across frequency band. Then we set the Type I error rate to 0.01, which equated to 53 time bins that could have been significant by chance (0.01 ×  × 38 frequency bins = 53 time bins). The *t *score from the pseudopopulation that corresponded to this Type I error rate was compared to that obtained from the original dataset at a given electrode and frequency band; if the *t *score from the original dataset was more significant than the *t *score generated by the pseudopopulation, we considered the original dataset to be significant (randomization test, *p *< 0.01, *df *= 11).

## Declaration of Competing interests

The author(s) declare that they have no competing interests.

## Authors' contributions

GH designed and assembled the EEG system, collected data, performed all analyses, developed analysis techniques (e.g., Z-transform to baseline and paired randomization test procedure), and wrote the manuscript. JJ performed preliminary data analyses and wrote the hardware driver code for realtime interface. AG wrote the C code that generated the Sternberg experiment and the grating stimuli, and assisted with data collection. JD assisted with data collection and preliminary behavioral analysis. RS provided technical input in the design of the grating task. MJK conceived the experiment and contributed important insights to the manuscript. All authors read, provided feedback, and approved the manuscript.
